# crossnma: An R package to synthesize cross-design evidence and cross-format data using network meta-analysis and network meta-regression

**DOI:** 10.1186/s12874-023-02130-0

**Published:** 2024-08-05

**Authors:** Tasnim Hamza, Guido Schwarzer, Georgia Salanti

**Affiliations:** 1 Institute of Social and Preventive Medicine, University of Bern, Bern, Switzerland; 2 Graduate School for Health Sciences, University of Bern, Bern, Switzerland; 3 Institute of Medical Biometry and Statistics, Faculty of Medicine and Medical Center, University of Freiburg, Freiburg, Germany

**Keywords:** R package, Network meta-analysis, Network meta-regression, Real-world evidence, Observational studies, Risk of bias

## Abstract

**Background:**

Although aggregate data (AD) from randomised clinical trials (RCTs) are used in the majority of network meta-analyses (NMAs), other study designs (e.g., cohort studies and other non-randomised studies, NRS) can be informative about relative treatment effects. The individual participant data (IPD) of the study, when available, are preferred to AD for adjusting for important participant characteristics and to better handle heterogeneity and inconsistency in the network.

**Results:**

We developed the R package **crossnma** to perform cross-format (IPD and AD) and cross-design (RCT and NRS) NMA and network meta-regression (NMR). The models are implemented as Bayesian three-level hierarchical models using Just Another Gibbs Sampler (JAGS) software within the R environment. The R package **crossnma** includes functions to automatically create the JAGS model, reformat the data (based on user input), assess convergence and summarize the results. We demonstrate the workflow within **crossnma** by using a network of six trials comparing four treatments.

**Conclusions:**

The R package **crossnma** enables the user to perform NMA and NMR with different data types in a Bayesian framework and facilitates the inclusion of all types of evidence recognising differences in risk of bias.

**Supplementary Information:**

The online version contains supplementary material available at 10.1186/s12874-023-02130-0.

## Background

### Background to network meta-analysis

Studies that estimate treatment effects are identified, evaluated, and synthesized in systematic reviews to obtain evidence that answers treatment-related questions [1]. Systematic reviews may include a pairwise meta-analysis (PMA) [2, 3], which is a statistical summary of findings from multiple studies comparing two interventions. The PMA extends to network meta-analysis (NMA) to compare multiple competing interventions, providing estimates for the relative effects of each pair of competing treatments [4]. The final NMA estimates are a combination of direct estimates derived from combining study findings and indirect estimates obtained using one or more intermediate comparators under the consistency assumption.

Most NMAs use aggregate data (AD) obtained from published studies. To explore between-study heterogeneity and between-comparison inconsistency in NMA, we need to study the role of important patient- and study characteristics, typically in subgroup analyses or meta-regressions [5]. As relationships at study level often fail to reflect associations at individual patient level, aggregated data are not suitable to explore the role of patient-level characteristics in modifying the treatment effects [6]. However, retrieving individual participant data (IPD) is a difficult and time-consuming endeavour. The most common scenario is to obtain IPD of some of the included studies, and then combine IPD and AD in a single model.

The vast majority of published NMAs synthesise data from randomised clinical trials (RCTs). Although RCTs are by design less prone to selection bias than non-randomised designs, biases can still arise from in their conduct [7–9] and reported findings [10] or from conflict of interest that can distort the body of evidence [11]. Generalisability of their findings to inform clinical practice is challenged by several of their features: RCTs include patients not necessarily representative of those encountered at the point of care, they are more likely to use placebo or other legacy treatments which are not an option in practice [10–12], and they often do not provide data on long-term benefit and safety of interventions. Pragmatic trials offer an alternative, but they can also be costly and difficult to conduct to study rare conditions, so that randomized evidence can be sparse in some health research fields. For these reasons, researchers on evidence synthesis consider sometimes evidence from non-randomised studies (NRS) despite the risk of confounding and several other biases inherent in their design. To assess the risk of bias (RoB) in the results of a study, tools have been created: the ROBINS-I for NRS [13] and the RoB 2 for RCT [14].

To handle different types of studies and data types, we recently introduced a suite of four Bayesian NMA and network meta-regression (NMR) models [15] extended a previously described three-level hierarchical model that combines IPD and AD [16–18] and included methods to combine these data when they come from RCT and NRS. The four models can be broadly described as an unadjusted (“naïve”) NMA model, where differences in bias between different study designs are ignored; a model that synthesises only RCT data with prior distributions for the relative treatment effects constructed from NRS evidence and potentially discounted according to RoB; and two bias-adjusted models where the relative treatment effect of each study is adjusted according to the underlying risk of bias.

We have implemented the different cross-NMA models in a new R package called **crossnma** (**cross**-design and **cross**-format **n**etwork **m**eta-**a**nalysis and network meta-regression). The package enables researchers to perform NMA and NMR on data that are available in different formats as IPD, AD or a combination of both, and each format can come from different study designs as RCT, NRS, or a mixture of both.

This work has been done within the HTx Horizon 2020 project. HTx is supported by the European Union, lasting for 5 years from January 2019. The main aim of HTx is to create a framework for the Next Generation Health Technology Assessment (HTA) to support patient-centered, societally oriented, real-time decision-making on access to and reimbursement for health technologies throughout Europe.

### Synthesis models available in **crossnma**

Below we provide a brief description of the four NMR models implemented in **crossnma**; NMA models can be obtained by simply ignoring covariate terms. Of note, the NMR model is described with one covariate for simplicity and ease of explanation. However, our **crossnma** package is designed to handle up to three covariates. The model used in the analysis is defined in R function *crossnma.model().* The notation used is summarized in Table 1. More details are available in the methodological publication [15].

**Table 1:** Notations used in synthesis models

Notation	Description	Argument in *crossnma.model()*
$i$	participant id	
$j$	study id	*study*
$k$	treatment index	*trt*
${y}_{ijk}$	outcome (IPD)	*outcome*
${y}_{jk}$	outcome (AD)	*outcome*
$b$	study-specific reference / baseline	
A	network reference	*reference*
${x}_{ijk}$	covariate (IPD)	*cov1, cov2, cov3*
${\overline{x}}_{j}$	mean covariate for study $j$ (AD)	*cov1, cov2, cov3*
${z}_{j}$	study characteristic to estimate the bias probability ${\pi }_{j}$	*bias.covariate*
$w$	common variance inflation factor for NRS estimates	*run.nrs.var.infl*
$\zeta$	common mean shift of NRS estimates	*run.nrs.mean.shift*

#### Unadjusted synthesis of data from RCTs and NRS 

This analysis combines the RCT and NRS evidence without accounting for the fact that different studies pertain to different risk of bias (argument *method.bias* = *"naive"* in *crossnma.model()*). Data from each study can be available as IPD or AD. The different types of studies and data format are combined using a three-level hierarchical model.

##### Level 1 (individual participant level)

When IPD is available, we observe the outcome ${y}_{ijk}$ for participant $i$ in study $j$ receiving treatment $k$. Each study has a reference treatment $b$. Observed outcomes are assigned an appropriate likelihood distribution with unknown parameters ${\varphi }_{ijk}$. Then, using a link function $g(.)$, these parameters are linked to the treatment and covariate effects. The distribution and link function in the model depend on the nature of the data and the effect measure we want to estimate. For example, when we observe binary data and want to estimate odds ratios, a Bernoulli likelihood is considered for the outcome and $g(.)$ is the logit function. The general form of the NMR model is

$$g\left({\varphi }_{ijk}\right)={u}_{jb} + {\delta }_{jbk}+{{\beta }_{0j} x}_{ijk}+{\beta }_{1bk}^{W}{x}_{ijk}+({\beta }_{1bk}^{B}-{\beta }_{1bk}^{W}) {\overline{x} }_{j.}$$

where ${x}_{ijk}$ and ${\overline{x} }_{j}$ are covariate value and its study mean, respectively. The prognostic effect of the covariate is quantified by ${\beta }_{0j}$; ${\beta }_{1bk}^{B}$ is the between-study interaction effect, which represents the associations between treatment and study’s mean covariate, and ${\beta }_{1bk}^{W}$ quantifies the within-study interactions between treatment effect and covariate. When ${x}_{ijk}={\overline{x} }_{j}=0,$ ${u}_{jb}$ can be interpreted as the average $g(.)$-transformed outcome in the study reference arm $b$, and ${\delta }_{jbk}$ is a study-specific relative effect of treatment $k$ versus $b$.

##### Level 2 (study-level)

In AD studies, we observe the mean outcome per study arm, ${y}_{jk}$, which is also assigned an appropriate likelihood distribution with unknown parameter ${\varphi }_{.jk}$. Then this parameter is linked to the model parameters via the link function:

$$g\left({\varphi }_{.jk}\right)={u}_{jb} + {\delta }_{jbk}+{\beta }_{1bk}^{B} {\overline{x} }_{j.}$$

##### Level 3 (cross-studies synthesis)

We combine the parameters from different studies assuming either a random-effects or a common-effect model. Table 2 summarizes the different assumptions supported by **crossnma** along with corresponding arguments to set each assumption in the package. It is important to highlight that studies with AD do not contribute to the estimation of ${\beta }_{0j}$ and ${\beta }_{1bk}^{W}$.

**Table 2:** Assumptions of synthesis model parameters

Parameter	Assumptions	Argument in *crossnma.model()*
Relative treatment effect (${\delta }_{jbk}$)	Random-effects: ${\delta }_{jbk}\sim \mathrm{\rm N}({d}_{Ak}-{d}_{Ab},{\tau }^{2})$	*trt.effect = 'random'*
	Common-effect: ${\delta }_{jbk}={d}_{Ak}-{d}_{Ab}$	*trt.effect = 'common'*
Covariate effect (${\beta }_{0j}$)	Independent effects: ${\beta }_{0j}\sim \mathrm{\rm N}(0,{\left\{15*{ML}_{max}\right\}}^{2} )$	*reg0.effect = 'independent'*
	Random-effects: ${\beta }_{0j}\sim \mathrm{\rm N}({B}_{0},{\tau }_{0}^{2})$	*reg0.effect = 'random'*
Within-study covariate-treatment interaction (${\beta }_{1,jbk}^{W}$)	Independent effects: ${\beta }_{1bk}^{W}\sim N(0,{\left\{15*{ML}_{max}\right\}}^{2} )$	*regw.effect = 'independent'*
	Random-effects: ${\beta }_{1bk}^{W}\sim \mathrm{\rm N}({B}_{1Ak}^{W}-{B}_{1Ab}^{W},{\tau }_{W}^{2})$	*regw.effect = 'random'*
	Common-effect: ${\beta }_{1j}^{W}={B}_{1}^{W}$	*regw.effect = 'common'*
Between-study covariate-treatment interaction (${\beta }_{1,jbk}^{B}$)	Independent effects: ${\beta }_{1j}^{B}\sim \mathrm{\rm N}(0,{\left\{15*{ML}_{max}\right\}}^{2} )$	*regb.effect = 'independent'*
	Random-effects: ${\beta }_{1j}^{B}\sim \mathrm{\rm N}({B}_{1Ak}^{B}-{B}_{1Ab}^{B},{\tau }_{B}^{2})$	*regb.effect = 'random'*
	Common-effect: ${\beta }_{1bk}^{B}={B}_{1}^{B}$	*regb.effect = 'common'*
Bias effect (${\gamma }_{m,jbk}$), $m=\mathrm{1,2}$	Random-effects: ${\gamma }_{m,jbk}\sim \mathrm{\rm N}({g}_{m,bk},{\tau }_{m,\gamma }^{2})$	*bias.effect = 'random'*
	Common-effect: ${\gamma }_{m,jbk}={g}_{m,bk}$	*bias.effect = 'common'*
Mean bias effect ${g}_{m,bk}$	The treatment $k$ is active. ${g}_{m,bk}={g}_{m}$ ($b$ inactive), ${g}_{m,bk}=0$ ($b$ active & no bias) ${g}_{m,bk}={g}_{m}^{act}$($b$ active & bias)	*unfav, bias.group*
Bias probability (${\pi }_{j}$)	${\pi }_{j}\sim Beta({a}_{1},{a}_{2})$	*pi.high.nrs,pi.low.nrs,pi.high.rct,pi.low.rct*
	$logit({\pi }_{j})=e+f{z}_{j}$	*bias.covariate*

Random-effects model assumes treatment/covariate effects vary across studies

Common-effect model assumes a single treatment/covariate effect shared by all studies

Independent effects model considers covariate effects in studies independently

By default, we assign minimally informative prior distributions to the main parameters following Valkenhoef et al. [19]: normal distribution $\mathrm{\rm N}(0,{\left\{15*{ML}_{max}\right\}}^{2} )$ for ${u}_{jb},{\beta }_{0j}, {B}_{0}, {B}_{1}^{W},{B}_{1}^{B},{d}_{Ak}$ and uniform distribution ${\text{Unif}}(0, {ML}_{max})$ for $\tau ,{\tau }_{{B}_{0}},{\tau }_{B},{\tau }_{W}$. The quantity ${ML}_{max}$ is calculated by first computing the maximum likelihood estimate of the relative treatment effect for each study and then taking the maximum value of these estimates. Users can provide their own prior distribution for the heterogeneity standard deviation parameters $\tau ,{\tau }_{{B}_{0}},{\tau }_{B},{\tau }_{W}$.

#### Use NRS priors for basic parameters in RCT model

In this model, we use NMR to estimate the relative treatment effects using only the NRS (argument *method.bias* = *"prior"* in *crossnma.model();* arguments starting with *run.nrs* can be used to control this process). We use the mean ${d}_{Ak}^{NRS}$ and variance ${V}_{AK}^{NRS}$ of the NRS posterior distribution of the relative treatment effects to construct prior distributions for the treatment effects and fit the model in the RCT data; ${{d}_{Ak}\sim \mathrm{\rm N}(d}_{Ak}^{NRS},{V}_{AK}^{NRS})$. To limit the impact of NRS on RCT estimates, we can either inflate the prior variance by dividing it by a common inflation factor $w$ with $0<w<1$ or shift NRS means by $\zeta$ (see reference [20] for more discussion on how to set $\zeta$ or reference [21] to choose a value based on elicited expert opinion). Treatment effects not observed in NRS are given the default priors; ${d}_{Ak}\sim \mathrm{\rm N}(0,{\left\{15*{ML}_{max}\right\}}^{2} )$.

#### Bias-adjusted model 1

In this and the next model, we adjust treatment effects according to each study’s RoB [22]. The level 1 model in Unadjusted synthesis of data from RCTs and NRS section is extended for bias-adjusted model 1 (*method.bias = "adjust1"*) is extended as follows (additional terms are printed in bold):

$$g\left({\varphi }_{ijk}\right)={u}_{jb} + {{\varvec{\delta}}}_{{\varvec{j}}{\varvec{b}}{\varvec{k}}}{{\varvec{\gamma}}}_{1,{\varvec{j}}{\varvec{b}}{\varvec{k}}}^{{{\varvec{R}}}_{{\varvec{j}}}}+{{\varvec{\delta}}}_{{\varvec{j}}{\varvec{b}}{\varvec{k}}}+{{{\varvec{\gamma}}}_{2,{\varvec{j}}{\varvec{b}}{\varvec{k}}}{\varvec{R}}}_{j}+{{\beta }_{0j} x}_{ijk}+{\beta }_{1bk}^{W}{x}_{ijk}+({\beta }_{1bk}^{B}-{\beta }_{1bk}^{W}) {\overline{x} }_{j}$$

and the level 2 model becomes $g\left({\varphi }_{.jk}\right)={u}_{jb} + {{\varvec{\delta}}}_{{\varvec{j}}{\varvec{b}}{\varvec{k}}}{{\varvec{\gamma}}}_{1,{\varvec{j}}{\varvec{b}}{\varvec{k}}}^{{{\varvec{R}}}_{{\varvec{j}}}}+{{\varvec{\delta}}}_{{\varvec{j}}{\varvec{b}}{\varvec{k}}}+{{{\varvec{\gamma}}}_{2,{\varvec{j}}{\varvec{b}}{\varvec{k}}}{\varvec{R}}}_{{\varvec{j}}}+{\beta }_{1bk}^{B} {\overline{x} }_{j.}$

${R}_{j}$ is sampled from a Bernoulli distribution with bias probability ${\pi }_{j}$_,_ for each study. Then ${\pi }_{j}$ is assigned a beta distribution; ${\pi }_{j}\sim Beta\left({a}_{1},{a}_{2}\right)$ where the values of ${a}_{1}$ and ${a}_{2}$ reflect the RoB (low, high or unclear) within RCTs or NRS. The ratio ${a}_{1}/{a}_{2}$ controls the skewness of the beta distribution. When the ratio ${a}_{1}/{a}_{2}$ approaches 1, the mean probability of bias gets closer to 1 and the study acquires ’major’ bias adjustment. The default beta priors in the package are: high bias RCT *prior.pi.high.rct* = *"dbeta(10,1)"*, low bias RCT *prior.pi.low.rct* = *"dbeta(1,10)"*, high bias NRS *prior.pi.high.nrs* = *"dbeta(30,1)"* and low bias NRS *prior.pi.low.nrs* = *"dbeta(1,30)"*. Alternatively, we can use study characteristics ${z}_{j}$ to predict ${\pi }_{j}$ through a logistic transformation (internally coded); $logit({\pi }_{j})=e+f{z}_{j}$. When ${z}_{j}$ is a continuous outcome, ${\text{exp}}(e)$ is the odds of bias at ${z}_{j}=0$ and ${\text{exp}}(f)$ is the odds ratio of bias for a one unit increase in ${z}_{j}$. When $f$ has a positive value, the bias probability increases with increasing values of ${z}_{j}$.

Table 2 shows how we combine the multiplicative ${(\gamma }_{1,jbk})$ and the additive (${\gamma }_{2,jbk})$ treatment-specific bias effects across studies using random-effects or common-effect models.

#### Bias-adjusted model 2

Another way to account for differences in RoB of the studies is to replace ${\delta }_{jbk}$ with a bias-adjusted relative treatment effect ${\theta }_{jbk}$ (*method.bias* = *"adjust2"*) [23].

The equations become for level 1 and 2 are

$$g\left({\varphi }_{ijk}\right)={u}_{jb} + {{\varvec{\theta}}}_{{\varvec{j}}{\varvec{b}}{\varvec{k}}}+{{\beta }_{0j} x}_{ijk}+{\beta }_{1bk}^{W}{x}_{ijk}+({\beta }_{1bk}^{B}-{\beta }_{1bk}^{W}) {\overline{x} }_{j.}$$

and

$$g\left({\varphi }_{.jk}\right)={u}_{jb} + {{\varvec{\theta}}}_{{\varvec{j}}{\varvec{b}}{\varvec{k}}}+{\beta }_{1bk}^{B} {\overline{x} }_{j}.$$

Then ${\theta }_{jbk}$ is given either a random-effect bimodal normal distribution; ${\theta }_{jbk}\sim \left(1-{\pi }_{j}\right)\mathrm{\rm N}\left({d}_{Ak}-{d}_{Ab},{\tau }^{2}\right)+{\pi }_{j}\mathrm{\rm N}\left({d}_{Ak}-{d}_{Ab}+{\gamma }_{jbk},{\tau }^{2}+{\tau }_{\gamma }^{2}\right)$ or assumed common across studies; ${\theta }_{jbk}={d}_{Ak}-{d}_{Ab}+{\pi }_{j}{\gamma }_{jbk}.$

The likelihood of the unknown parameter ${\theta }_{jbk}$ is

$$L\left({\theta }_{jbk}; {d}_{Ak},{d}_{Ab},\tau ,{\tau }_{\gamma },{\pi }_{j}\right)=\frac{\left(1-{\pi }_{j}\right)}{\sqrt{2\pi {\tau }^{2}}}{\text{exp}}\left\{-\frac{{\left({\theta }_{jbk}-\left({d}_{Ak}-{d}_{Ab}\right)\right)}^{2} }{2{\tau }^{2}}\right\}+\frac{{\pi }_{j}}{\sqrt{2\pi \left({\tau }^{2}+{\tau }_{\gamma }^{2}\right)}}{\text{exp}}\left\{-\frac{{\left({\theta }_{jbk}-\left({d}_{Ak}-{d}_{Ab}+{\gamma }_{jbk}\right)\right)}^{2} }{2\left({\tau }^{2}+{\tau }_{\gamma }^{2}\right)}\right\}.$$

Here, the bias probability ${\pi }_{j}$ determines the weight of the bias-adjusted distribution (second part of the equation) in the overall likelihood $L\left({\theta }_{jbk}; {d}_{Ak},{d}_{Ab},\tau ,{\tau }_{\gamma },{\pi }_{j}\right)$. The term ${\gamma }_{jbk}$ is the bias effect, as in bias-adjusted model 1.

## Implementation

We implement the Bayesian models in a new R package called **crossnma**. The user can install the package with the command *install.packages("crossnma")* and then load the library into the current R session with *library("crossnma")*.

The Bayesian model is run in the background using Just Another Gibbs Sampler (JAGS) software [24]. Therefore, the JAGS programme must be installed on the user’s local computer (see https://sourceforge.net/projects/mcmc-jags/). A vignette with a binary data example is part of **crossnma** which can be opened using *vignette(“crossnma”)*. Package updates providing new features or fixing bugs will be posted on the package website: https://github.com/htx-r/crossnma.

### Workflow within **crossnma**

**Fig. 1:**
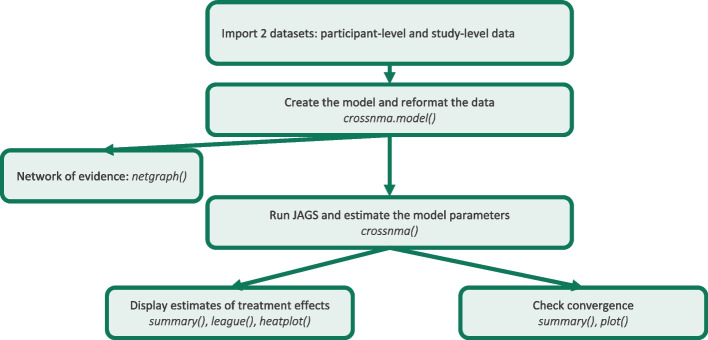
Workflow within the R package **crossnma**. The direction of arrow indicates a function’s output is used as input to another function

Figure 1 presents the workflow for conducting analyses within **crossnma**. Before running *crossnma()*, we display the network of evidence using *netgraph()* (which is a generic function in **netmeta**) to display the network of evidence. To conduct the data synthesis, there are two main steps: use *crossnma.model()* to produce the JAGS code and reformat the data, then pass the output to *crossnma()*, which matches the data with the model, runs the analysis and estimates all model parameters. The generic function *plot()* can produce a trace plot to evaluate the Markov chain Monte Carlo (MCMC) convergence for each model parameter. The functions *summary()*, *league()* and *heatplot()* can be used, with the output of *crossnma()* as input, to produce a numerical and graphical summaries of the treatment effect estimates. More details on how to use of the functions and their arguments can be found in Supplementary Document S1.

###  Comparison with some of the available packages

We compare the output of **crossnma (version 1.2.0)** with **BUGSnet** **(version 1.1.0)**, **gemtc (version 1.0.2)**, **multinma (version 1.1.2) bnma (version 1.5.1)** and **netmeta (version 2.8-2)** concerning various features in Table 3. Additionally, we assess the performance of some of these packages using a dataset provided by **crossnma**, which we describe in the next section. As **BUGSnet** and **gemtc** can only synthesize aggregate data, we summarize IPDs at the arm level (Supplementary Data S1). Then we perform NMA with a random-effects model using all packages. Treatment effect estimates (odds ratios) from **crossnma** and the three other packages do not differ beyond the MC error, however, the **BUGSnet** estimate of the between-study variance ($\tau$) is substantially larger compare to other packages. This can be attributed to the fact that **BUGSnet** uses an unrealistic default prior distribution $\sigma \sim Unif(\mathrm{0,1.62})$ where $\tau =1/{\sigma }^{2}$. In **crossnma**, we set $\tau \sim Unif(\mathrm{0,1.62})$. R code and detailed output of our analyses are provided in Supplementary Data S2, Supplementary Tables S1, S2, S3 and S4.

**Table 3:** A comparative overview of packages for (network) meta-analysis and network meta-regression with different criteria

Package/criteria	AD meta-analysis	IPD meta-analysis	AD meta-regression	IPD meta-regression	IPD+AD meta-analysis	IPD+AD meta-regression	Accounting for study risk of bias	Split the within- and between-study covariate coefficients	Same treatment effects in IPD and AD levels	Estimate AD treatment effect by integration over joint covariate distribution
crossnma	✔	✔	✔	✔	✔	✔	✔	✔	✔	
multinma	✔	✔	✔	✔	✔	✔				✔
BUGSnet	✔		✔							
gemtc	✔									
bipd		✔		✔						
netmeta	✔									

We performed NMR for age using **crossnma** and **multinma**. Supplementary Table S5 shows the estimated treatment effects. The disagreement in the estimation is due to the differences in the implemented models and variations in each package's built-in analysis settings. The code for conducting this comparative analysis is provided in Supplementary Data S4.

## Working example

In the following, we illustrate NMA and NMR in **crossnma**. We analyse fictitious data, simulated to mimic real RCTs with IPD and AD [25]. The code for each analysis is available in Supplementary Data S3 (as well as presented in the vignette).

### Description of the network

The evidence network consists of four drugs examined in six studies, with aggregate data (two RCTs) or individual participant data (three RCTs and one cohort study). The IPD dataset contains 1944 rows, i.e., study participants. The AD dataset is provided in arm-level format, with each row representing a study arm and the same variable names as the IPD dataset. Below, we present the two datasets: the first few rows of the IPD dataset and the complete set of rows for the AD dataset. We evaluate the treatment effect using a binary outcome of relapse after two years of follow-up. The relative treatment effects are expressed as odds ratios, where an OR below 1 indicates the treatment is preferable to the reference.
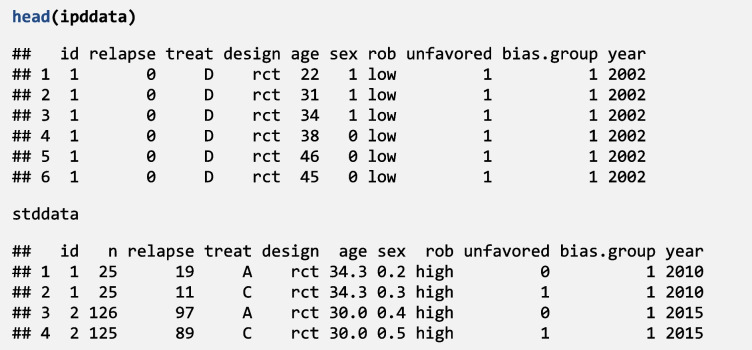


### Data synthesis using the four models available in **crossnma**

We continue with the analysis as the network is connected. We begin with creating a JAGS model and reformatting both datasets using *crossnma.model()*. Then, as the network is connected, the output of *crossnma.model()* is passed to *crossnma()*, which runs NMA with MCMC for 5000 iterations, 2000 burn-in, one thinning and two chains (default settings). In this example, we use drug A as a reference treatment except in the analysis in Using non-randomized studies as a prior in network meta-regression section drug D is the reference.

#### Unadjusted network meta-analysis

First, we synthesize data from RCT and NRS without distinguishing between them (method.bias = 'naive'). Because there are few studies in the network, we expect the heterogeneity parameter to be estimated inefficiently and thus assume a more informative prior to improve estimation, $\tau \sim N(0, 1/3)$. The data is analyzed using odds ratio as a summary measure sm = "OR"(which is the default for binary outcomes). By choosing trt.effect = 'random' (default), we are assigning a normal distribution to each relative treatment effect to allow the synthesis across studies, Table 2 lists all supported options.

We can also compute the values of Surface Under the Cumulative Ranking (SUCRA) (by enabling the sucra = TRUE option), but it's essential to specify a negative preferred direction for the outcome (using small.values = "desirable"). This setting indicates that lower values of the relative treatment effect signify the treatment's effectiveness. Conversely, if positive values are preferred, you can set small.values = "undesirable".
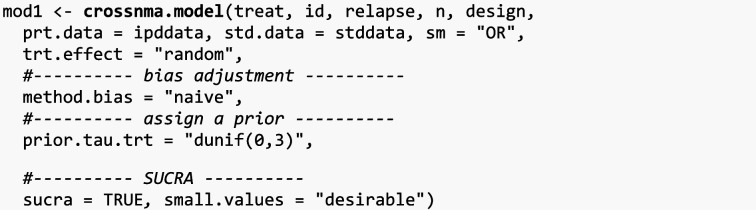


**Fig. 2:**
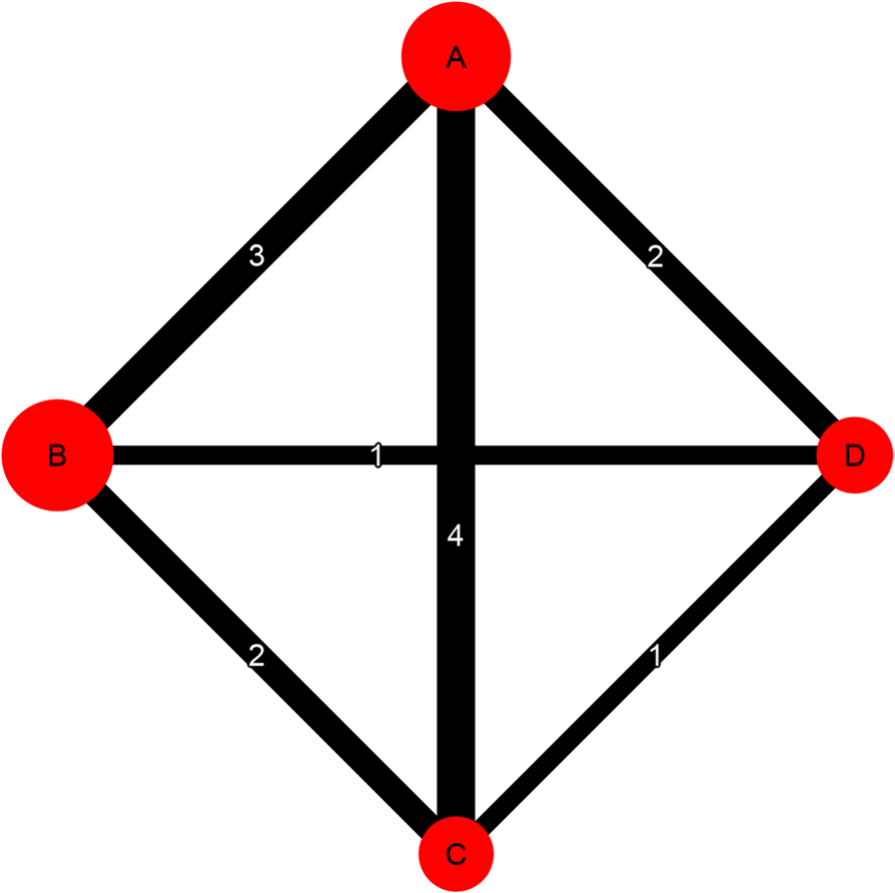
Network plot


The network graph in Fig. 2 was generated with the following command:

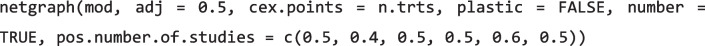


In the graph, the thickness of the edges corresponds to the number of studies, while the number of studies is displayed on the edges. Additionally, the node size reflects the number of participants who received each intervention.

Next, we fit the NMA model using *crossnma()* where we can set the number of iterations, burn-in, thinning and chains. We run all subsequent models under the same settings. Note that for our example, we run the MCMC with settings different from the default to ensure convergence.
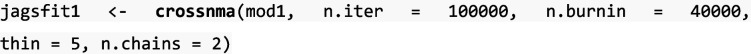


The R command *print(jagsfit1, backtransf = FALSE)* produces Table 4a which shows summary statistics for the results (Table 4b-e are also produced using the *print()* function for other models). The estimated OR of B vs A can be obtained as exp(d.B), and similarly exp(d.C) and exp(d.D) are the ORs of C and D relative to A.

**Table 4:** Summary statistics of estimates produced from the four models in **crossnma**, applied to the network presented in Fig. 2

	Mean	SD	2.5%	50%	97.5%	Rhat	n.eff
(a) Unadjusted network meta-analysis							
d.A	0	0	0	0	0	NaN	0
d.B	-0.766	0.214	-1.173	-0.77	-0.323	1.001	15,638
d.C	-0.467	0.229	-0.943	-0.459	-0.045	1.001	9265
d.D	-1.093	0.288	-1.665	-1.091	-0.528	1.001	12,634
tau	0.221	0.201	0.009	0.168	0.738	1.003	2896
SUCRA.A	0.007	0.051	0	0	0	1.011	20,158
SUCRA.B	0.678	0.162	0.333	0.667	1	1.001	15,492
SUCRA.C	0.375	0.14	0.333	0.333	0.667	1	16,108
SUCRA.D	0.941	0.151	0.333	1	1	1.001	11,897
(b) Unadjusted network meta-regression							
b_1	0.002	0.068	-0.104	0.003	0.106	1.001	45,520
d.A	0	0	0	0	0	NaN	0
d.B	-1.003	0.354	-1.699	-1.002	-0.307	1.004	1811
d.C	-0.492	0.394	-1.27	-0.493	0.272	1.001	1808
d.D	-1.039	0.513	-1.997	-1.054	0.021	1	1442
tau	0.225	0.199	0.007	0.174	0.764	1.001	4199
tau.b_1	0.056	0.102	0.001	0.024	0.349	1.002	1144
(c) Using non-randomized studies (NRS) to construct priors for randomized clinical trials model							
b_1	0.013	0.065	-0.076	0.013	0.102	1.059	10,998
d.D	0	0	0	0	0	NaN	0
d.A	0.954	0.375	0.222	0.949	1.699	1.001	2765
d.B	0.069	0.431	-0.769	0.063	0.931	1	3937
d.C	0.545	0.463	-0.342	0.54	1.474	1.001	3463
tau	0.323	0.275	0.011	0.246	1.055	1	4940
tau.b_1	0.045	0.096	0.001	0.017	0.297	1.075	961
(d) Bias-adjusted model 1							
d.A	0	0	0	0	0	NaN	0
d.B	-0.754	0.226	-1.178	-0.765	-0.266	1.016	15,190
d.C	-0.432	0.273	-0.991	-0.43	0.114	1.072	6478
d.D	-1.085	0.298	-1.675	-1.086	-0.491	1.006	16,110
g	-0.112	14.136	-32.759	-0.173	32.937	1.291	64,338
tau	0.235	0.211	0.008	0.177	0.809	1.036	3495
(e) Bias-adjusted model 2							
d.A	0	0	0	0	0	NaN	0
d.B	-0.767	0.287	-1.348	-0.767	-0.189	1	9205
d.C	-0.481	0.28	-1.096	-0.464	0.03	1.001	7780
d.D	-1.108	0.377	-1.899	-1.1	-0.382	1	9057
g	0.016	0.339	-0.626	0.003	0.723	1.002	5261
tau	0.294	0.248	0.01	0.229	0.962	1	1788

*Abbreviations*: *d.A, d.B d.Cand d.D* are the log odds ratios (${d}_{Ak}$) of each drug relative to the network reference (that is set D in (c) and A for the rest), *tau* is the heterogeneity standard deviation in treatment effect across studies $\tau$, *b_1* is the age effect (when ${B}_{1}^{W}={B}_{1}^{B}={B}_{1}$) and the heterogeneity standard deviation (${\tau }_{B1}={\tau }_{B}={\tau }_{W}$) of age effect across studies, *g* is the mean bias effect (${g}_{m}$), *Mean and SD* are the mean and the standard deviation of the posterior distribution, respectively, *2.5%, 50% and 97.5%* are the quantiles of the posterior distribution, *Rhat* is Gelman and Rubin statistic $\widehat{R}$, *n.eff* is the effective sample size

Function *print()* also produces the SUCRA rank estimates, where treatment D notably excels with the highest score of 0.941, signifying a strong likelihood of achieving favorable outcomes. In contrast, treatment A has the lowest score at 0.007, implying that it is the least likelihood choice for yielding positive outcomes.

#### Unadjusted network meta-regression

Next, we include age in the model as a potential effect modifier. Because we have few studies and little variation between them, we assume that the age effect within and between studies is equal (argument split.regcoef = FALSE).

In addition to relative treatment effects and its heterogeneity, we obtain estimates of the age effect (b_1 is ${B}_{1}^{W}={B}_{1}^{B}$) and the heterogeneity standard deviation (tau.b_1 is ${\tau }_{B}={\tau }_{W}$) in the effect of age across studies (Table 4b).
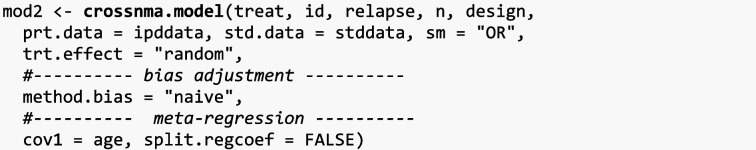


We could add two more covariates to the NMR model using arguments cov2 and cov3.

The MCMC is run under the same set up as in Unadjusted network meta-analysis section. The league table of the estimates of each treatment vs comparator can be shown as follows (we just display the first two lines of the output).
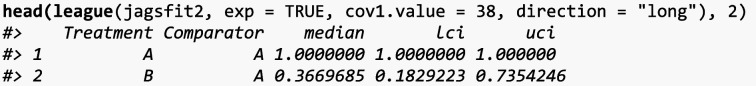


#### Using non-randomized studies as a prior in network meta-regression

We use the single NRS study to construct priors for a subsequent NMA fitted on RCT data. The prior variance is inflated by 60% to reduce the contribution of NRS on the final estimation ($w=0.6$). Table 4c presents the estimates where the reference treatment is drug D as drug A is not examined in NRS.
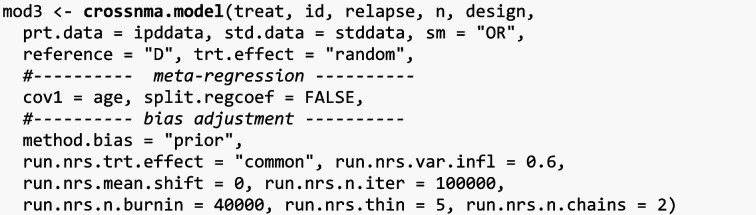


**Fig. 3:**
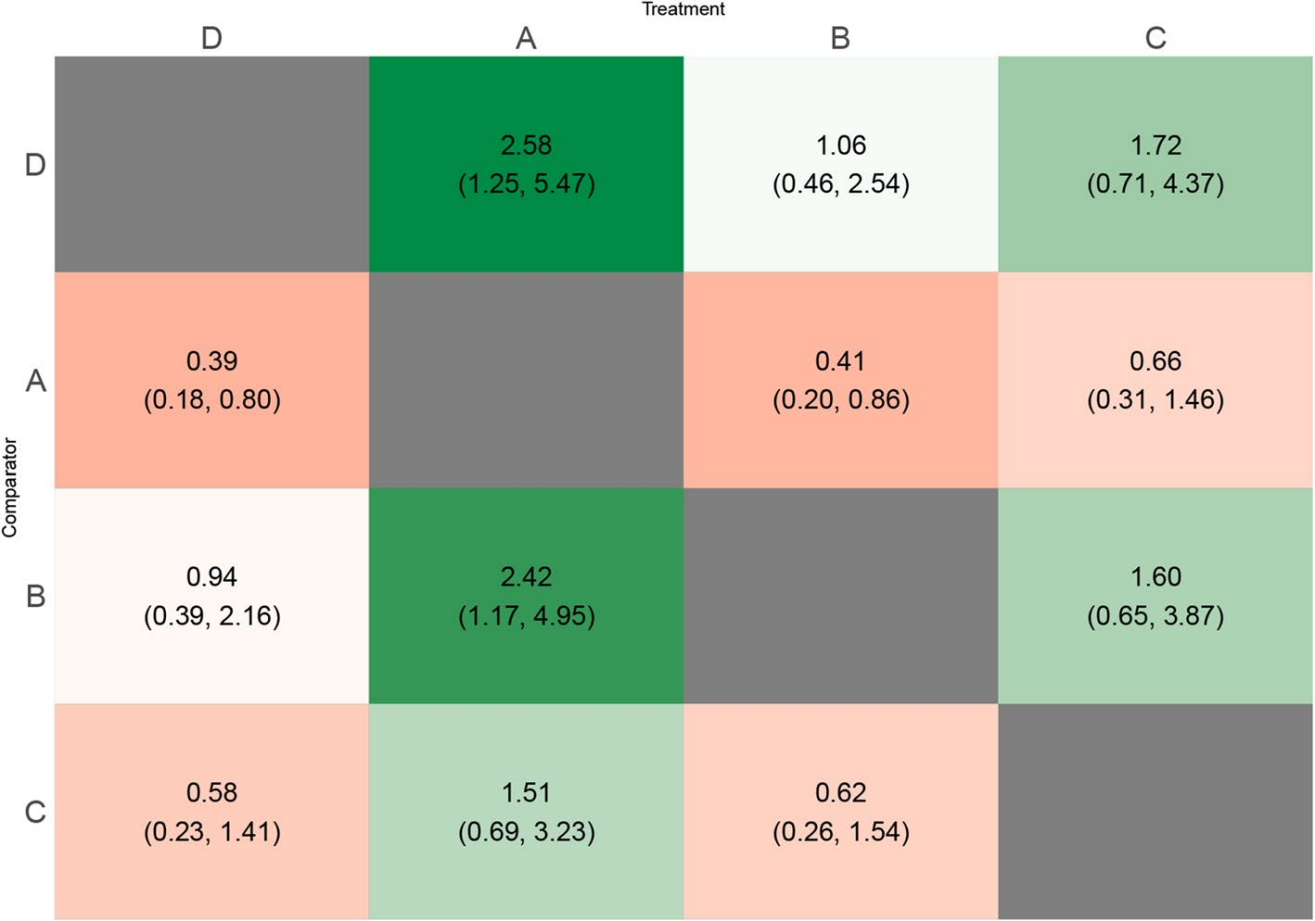
League table heatmap of relapse odds ratio (and 95% credible intervals) of the treatment on the top vs treatment on the left when the network (shown in Fig. 2) is analysed


The heat plot (Fig. 3) summarizes the relative effect with the 95% credible interval of each treatment on the top compared to the treatment on the left. All estimates are computed for participant age 38.

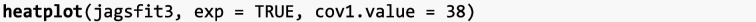


#### Bias-adjusted model 1


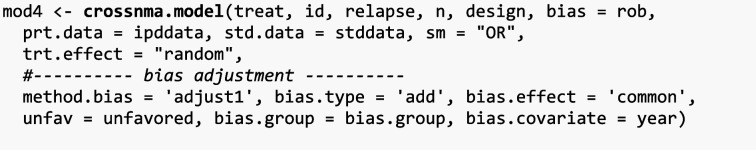


To fit this model, data needs to include study-level RoB data and indicate the direction of bias (which treatment in the study is expected to be favoured). We assume that additive bias effects are equal across studies. We estimate the probability of bias using the year of study publication.

The common bias effect (g (R output)) is estimated to be -0.112, indicating that older studies tend to overestimate the relative treatment effect when compared to new studies (see Table 4d). We note that we obtained very uncertain estimates for the mean bias effect. This is because the dataset includes only three studies at low and three studies at high RoB. In the presence of more studies, estimation improves both in convergence and precision as shown in Hamza et al. [26].

#### Bias-adjusted model 2

This analysis requires data similar to bias-adjusted model 1. We use the default beta priors to estimate the bias probabilities. The overall bias effect (g (R output)=${g}_{m}$ (model description)) is estimated to be 0.016 (Table 4e), implying that studies with a high RoB slightly underestimate treatment effect when compared to studies with a low RoB but this estimate again comes with large uncertainty.
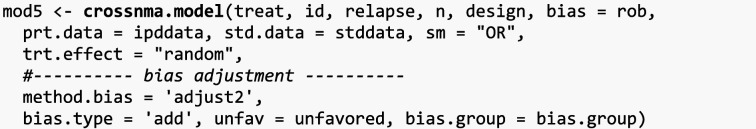


#### Models convergence

**Fig. 4:**
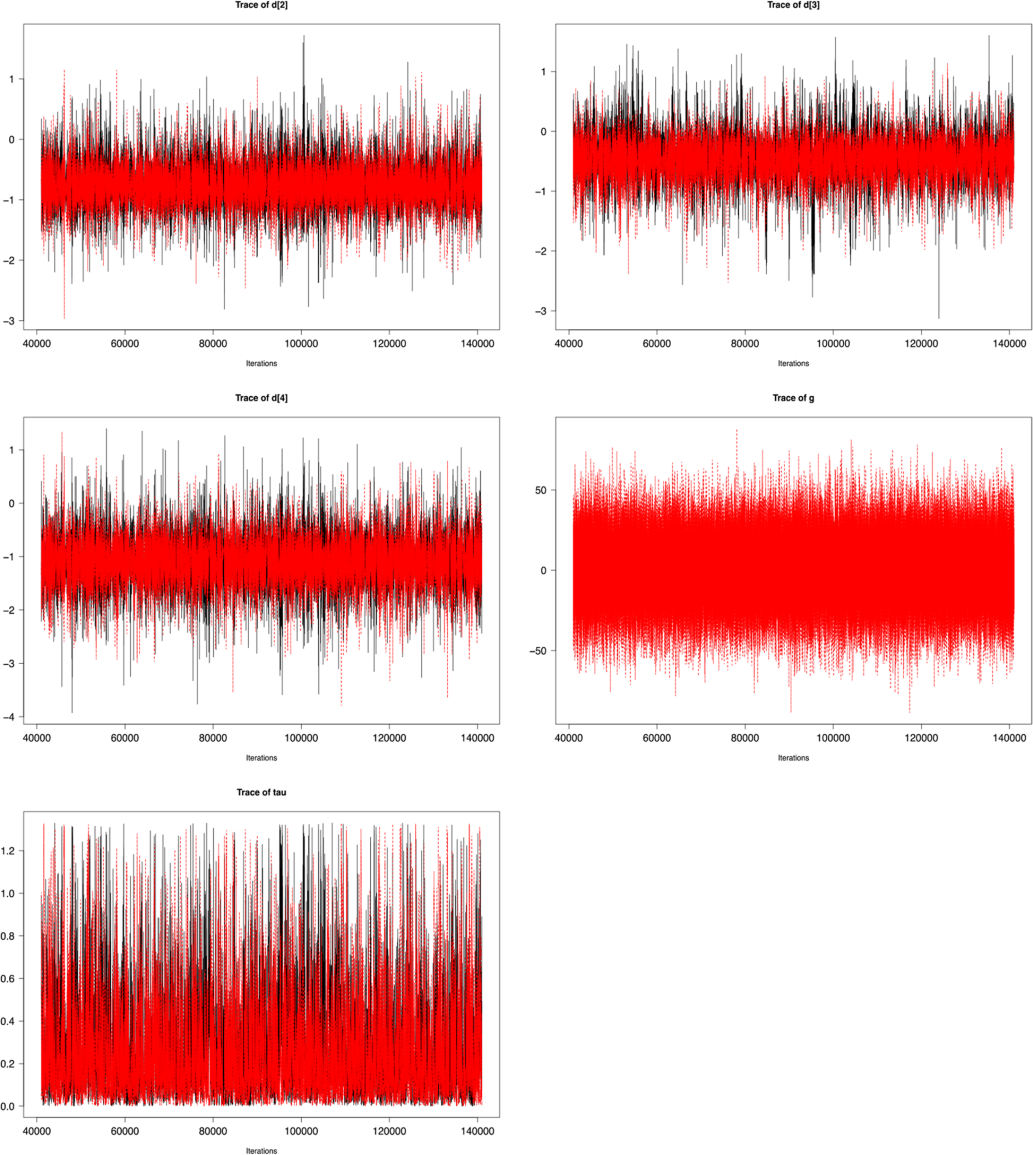
Trace plots of MCMC chains for the four basic parameters (${d}_{Ak}$) and the heterogeneity standard deviation ($\tau )$ and the mean bias effect ($g$) in bias-adjusted model 1 of network meta-analysis with the four treatments displayed in Fig. 2

To evaluate the convergence of the MCMC chains of all models, we use the Gelman and Rubin statistic $\widehat{R}$ and the number of effective sample sizes (n.eff) shown in Table 4 [27]. Except for the common bias effect (g (R output)) in bias-adjusted model 1, $\widehat{R}$ values are approximately 1. The values of n.eff indicates that sufficient independent samples are used to generate the final estimates. We inspect the trace plot (generated by *plot(*jagsfit4*)*) in Fig. 4 to further investigate the convergence of g (besides other parameters of the bias-adjusted model 1), and we observe a great deviation between samples. This is because the bias-adjusted model 1 includes the bias as a dichotomous variable, which requires having sufficient data at both low and high RoB. The dataset we analyze does not contain enough of this data (3 studies at low and 3 at high RoB).

**Fig. 5:**
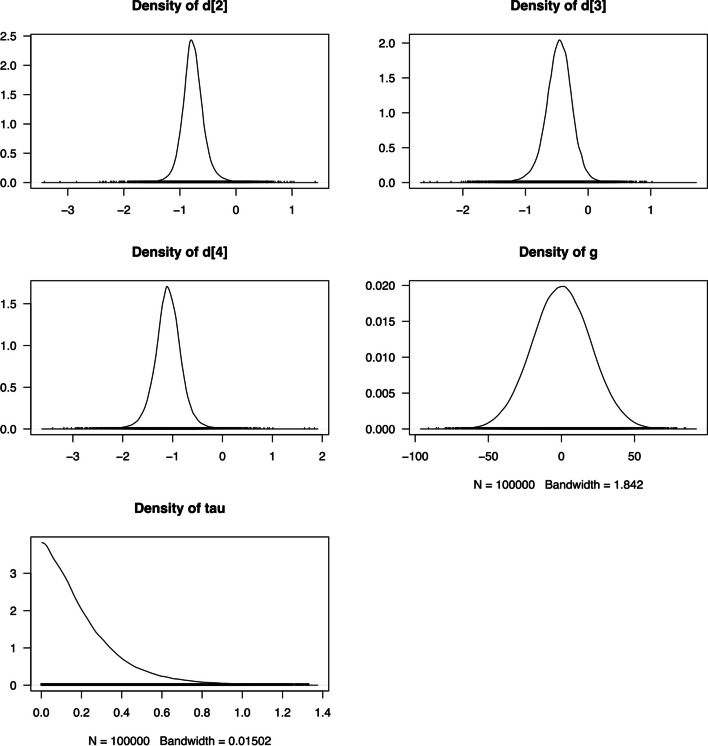
Density plots depicting the distributions of variables from the MCMC samples. This plot is generated for the four basic parameters (${d}_{Ak}$) and the heterogeneity standard deviation (τ) and the mean bias effect (g) in bias-adjusted model 1 of network meta-analysis with the four treatments displayed in Fig. 2

In Fig. 5, we present density plots to visualize the distributions of the variables derived from the MCMC samples. The density plots illustrate that most variables exhibit normal distributions, reflecting symmetrical data with a clear central tendency. However, in the case of $\tau$, a positive-only normal distribution is observed, indicating values restricted to the positive range. Notably, the density distribution of $g$ demonstrates a wide range of values, suggesting insufficient data for precise estimation. This finding underscores the importance of acquiring additional data to improve the accuracy and reliability of the estimation process.

#### Computational efficiency of **crossnma**

The five analyses were conducted using the **crossnma** package (version 1.2.0) in R (version 4.2.3) on a MacBook Pro (13-inch, 2019, Two Thunderbolt 3 ports) with a 1.4 GHz Quad-Core Intel Core i5 processor and 8 GB 2133 MHz LPDDR3 RAM. All analyses include 4 studies with IPD and 1944 participants and 2 studies with AD and 4 treatment arms, with a total of 4 treatments. The runtime of the analyses done in this article varied between 2 to 5 min, depending on the specific analysis. The longest runtime of 5 min was observed for network meta-regressions that included a single covariate (Unadjusted network meta-regression and Using non-randomized studies as a prior in network meta-regression sections). The two bias-adjustment models (Bias-adjusted model 1 and Bias-adjusted model 2 sections) took approximately 3 and a half minutes. The shortest runtime of 2 min was observed for NMA (Unadjusted network meta-analysis section) without adjustment for bias. For all analyses we run 100,000 iterations with a burn-in of 40,000 and thinning of 5 to ensure convergence.

## Discussion

In this paper, we introduce **crossnma***,* an R package that performs Bayesian NMA and NMR using the JAGS software. In **crossnma***,* data can be collected from different study designs, as RCT or NRS, and provided in IPD or AD formats. The functions within the package enable analysis, result representation and convergence evaluation. We provide detailed instructions on how to use **crossnma** and we demonstrate this with several analytic examples.

Several R packages for performing NMA on aggregate data are available, such as **gemtc** [19], **bnma** [28] and **BUGSnet** [29] in a Bayesian setting, or **netmeta** [30] under a frequentist framework. However, data is increasingly becoming available in a variety of formats and designs. For example, there is a growing in the number of IPD analyses, and only user-written code can be used to perform such analyses. The number of reviews that combine NRS and RCT data is rising as well, and unadjusted synthesis is widely used due to its simplicity and ease of implementation [31]. Network meta-regression using only aggregated data can be performed with **bnma**, **rnmamod**, **gemtc** and **BUGSnet**. Using the methodologies presented by Philippo et al. [32] the R package **multinma** models jointly effects estimated in studies with IPD and AD formats. The package **crossnma** implemnets another methodology to merge estimates both formats [26]. Furthermore, **crossnma** can perform sensitivity analyses for study-level bias in RCT and NRS data [32]. Some functions in **crossnma** are similar to and inspired by the Bayesian NMA packages **gemtc** and **BUGSnet**. In addition to handling both AD and IPD, the **crossnma** package can be used to account for various levels of study risk of bias and their impact in the results.

In **crossnma**, we implemented, among others, a model for the synthesis of IPD and AD data previously described in [16, 17, 26] assuming that the relative treatment effect in IPD and AD synthesis models are the same after accounting for effect modifiers and prognostic factors. A method that makes less assumptions and with theoretical advantages has been presented in [32]. Future updates of **crossnma** could include an option of the model currently implemented in [33].

Regarding the inclusion of NRS data, it is important to note that in situations where RCT data is unavailable for certain comparisons or treatment interventions, researchers may have to rely on NRS data to inform the analysis. However, it is crucial to recognize that NRS data are susceptible to various biases of unknown magnitude, which necessitates careful consideration when utilizing them in the analysis. In the **crossnma** package we implemented methods that can decrease the impact that such biases may have in the final NMA results.

While **crossnma** allows effect modifying covariates to work in a range of ways, including a different regression coefficient for each treatment, data might not enable their estimation. Hence, in practice users are more likely to employ the model that assumes the same regression coefficient for all treatments.

Several limitations should be acknowledged regarding the statistical approaches implemented in **crossnma**. First, incorporating NRS evidence as prior in the analysis of RCTs can be complicated in practice. Collecting expert opinion about the bias in NRS is time consuming and often impractical. The use of priors from NRS should be implemented via a sensitivity analysis using a range of “downweighing” values for the impact of the prior in the results of NMA. Second, the model by Verde includes a parameter for the probability of bias, which is difficult to estimate from the current data, so informative priors are required. To establish these priors as subjectively as possible, trained data extractors are needed to evaluate the risk of bias in each study using established and reproducible tools, like RoB-2 and ROBINS-I. Third, the identifiability of all model parameters, and in particular those that relate to bias, depend on the available data. Fourth, sensitivity to the choice of prior distribution necessitates conducting thorough sensitivity analysis. While we provide recommendations in our recent paper [26], further research is needed to explore alternative methods and enhance the applicability of bias-adjustment techniques in decision-making contexts. Fifth, the implemented models for the synthesis of AD and IPD are an approximations of the model implemented in **multinma** [33], and their performance is unknown. These models may not be readily generalizable for time-to-event outcomes. Future updates to the package will incorporate the models described in [32], thereby overcoming these limitations. Finally, **crossnma** assumes similar relative treatment effects in IPD and AD, which holds true only for non-collapsible outcomes. However, for non-collapsible outcomes like logit, this assumption introduces aggregation bias [6].

The model to combine IPD and AD implemented in **crossnma** assumes distinct regression coefficients for interaction terms at the IPD and AD levels. In contrast, the integration approach implemented in **multinma** do not require AD-specific interaction terms, as these are inherently defined by the integration process. The models implemented in **crossnma** can be viewed as an approximation to the models by Philippo et al. [32] which have a theoretical advantage. However, application of the latter model requires additional data or assumptions to establish the correlation structure between covariates, which can be challenging in practice. A large-scale comparison of these two modelling approaches using realistic scenarios would shed more light to the impact of model misspecification, violation of model assumptions and extend of aggregation bias.

In addition to the foundational assumptions that underlie conventional meta-analysis (e.g., the assumption that treatment effects are generalizable across patients from the included trials) and the assumptions inherent in NMA (e.g., a connected network and consistency of effects), all meta-regression models assume the absence of unobserved effect modifiers [16–18, 22, 23]. IPD network meta-regression models rely on the assumption of conditional constancy of relative effects, which asserts that relative effects remain constant across different populations at specific levels of a set of covariates.

We acknowledge the following **crossnma** shortcomings. In addition to the current functions that generate league tables and summary statistics, we plan to develop new functions to present results for the following purposes: displaying the distribution of potential effect modifiers by study, treatment, or both; presenting SUCRA scores as plots and tables to enable ranking treatments, and producing a plot of the estimates of relative treatment effects at various covariate values (for NMR model). Our package supports binary and continuous outcomes, analysed in the vast majority of published NMAs [34]. Future updates will include count and time-to-event outcomes. Also, we plan to develop a separate vignette that focuses specifically on continuous outcomes. These additional features and resource will provide users with a more comprehensive understanding of the package's versatility and how it can be applied in various analysis scenarios. In terms of summary measure, **crossnma** enables expressing relative treatment effects in terms of odds ratio or risk ratio for binary data and mean difference or standardised mean difference for continuous outcomes.

The data in **crossnma** must be provided at the arm level which may require additional data manipulation. For example, contrast-level data can be transformed to the arm-level format using R function *longarm()* from R package **meta**. A future extension will expand this to provide contrast-level data directly. Methods for evaluating inconsistency, including node splitting and unrelated mean effect, are not yet implemented in **crossnma**. We intend to address these issues in upcoming version of **crossnma**.

The package **crossnma** should be used in conjunction with the technical article that describe the models, their assumptions, and limitations. The vignette accompanying the **crossnma** package but mainly the publication by Hamza et al. [26] contains useful information to enable users to set up models that sensibly reflect the nature of their data. Users are advised in particular to pay close attention to the assumptions behind the models, which are described in [26].

## Conclusions

The R package **crossnma** enables the user to perform NMA and NMR with different data types in a Bayesian framework and facilitates the inclusion of all types of evidence accounting for their differences in risk of bias.

## Availability and requirements

Project name: crossnma project

Project home page: https://github.com/htx-r/crossnma

Operating system(s): Any OS providing R and JAGS

Programming language: R

Other requirements: JAGS 4.3.0

License: GNU GPL-2 or higher versions

Any restrictions to use by non-academics: no restrictions

### Supplementary Information

**Additional file 1.**

**Additional file 2: Supplementary Data S1.** This dataset is formed by merging both Aggregate Data (AD) and Individual Participant Data (IPD) from crossnma. This dataset is employed in the R code found in Supplementary Data S2 to perform a comparative analysis among the packages BUGSnet, gemtc, multinma, and crossnma.

**Additional file 3.**

**Additional file 4: Supplementary Table S1.** Summary statistics of network meta-anaylsis estimates produced by BUGSnet package, applied to the data in Supplementary Data S1.

**Additional file 5: Supplementary Table S2.** Summary statistics of network meta-anaylsis estimates produced by crossnma package, applied to the data in Supplementary Data S1.

**Additional file 6: Supplementary Table S3.** Summary statistics of network meta-anaylsis estimates produced by gemtc package, applied to the data in Supplementary Data S1.

**Additional file 7: Supplementary Table S4.** Summary statistics of network meta-anaylsis estimates produced by multinma package, applied to the data in Supplementary Data S1.

**Additional file 8: Supplementary Table S5.** Summary statistics of network meta-regression estimates produced by multinma and crossnma packages. These estimates are based on data available in crossnma, utilizing both Individual Participant Data (IPD) and Aggregate Data (AD).

**Additional file 9.**

**Additional file 10.**

**Additional file 11.** Reviewer reports submitted for version 1.

## Data Availability

Project name: crossnma project Project home page: https://github.com/htx-r/crossnma Operating system(s): Any OS providing R and JAGS Programming language: R Other requirements: JAGS 4.3.0 License: GNU GPL-2 or higher versions Any restrictions to use by non-academics: no restrictions

## Abbreviations

NMA: Network Meta-Analysis
NMR: Network Meta-Regression;
IPD: Individual Participant Data
AD: Aggregate Data
RCT: Randomised clinical trials
NRS: Non-Randomised Studies
RoB: Risk of Bias
JAGS: Just Another Gibbs Sampler
MCMC: Markov chain Monte Carlo
