# Supplementary material for: crossnma: An R package to synthesize cross-design evidence and cross-format data using network meta-analysis and network meta-regression
Source: BMC Med Res Methodol. 2024 Aug 5;24:169. doi: 10.1186/s12874-023-02130-0 (PMC11299362; doi:10.1186/s12874-023-02130-0)
Supplement: Supplementary file 1 — Additional file 1. [file 12874_2023_2130_MOESM1_ESM.docx]

## Create the cross NMA/NMR model

The *crossnma.model()* function is called in R as follows

*crossnma.model <- function(trt,*

*study,*

*outcome,*

*n,*

*design,*

*se,*

*##*

*cov1 = NULL,*

*cov2 = NULL,*

*cov3 = NULL,*

*##*

*bias = NULL,*

*unfav = NULL,*

*bias.covariate = NULL,*

*bias.group = NULL,*

*##*

*prt.data = NULL,*

*std.data = NULL,*

*##*

*sm,*

*reference = NULL,*

*trt.effect = "random",*

*level.ma = gs("level.ma"),*

*## ---------- SUCRA score ----------*

*sucra = FALSE,*

*small.values = NULL,*

*cov1.value = NULL,*

*cov2.value = NULL,*

*cov3.value = NULL,*

*## ---------- meta regression ----------*

*cov1.ref = NULL,*

*cov2.ref = NULL,*

*cov3.ref = NULL,*

*reg0.effect = "independent",*

*regb.effect = "random",*

*regw.effect = "random",*

*split.regcoef = TRUE,*

*## ---------- bias adjustment ----------*

*method.bias = NULL,*

*bias.type = NULL,*

*bias.effect = "common",*

*down.wgt = NULL,*

*## ---------- prior ----------*

*prior.tau.trt = NULL,*

*prior.tau.reg0 = NULL,*

*prior.tau.regb = NULL,*

*prior.tau.regw = NULL,*

*prior.tau.bias = NULL,*

*prior.pi.high.rct = NULL,*

*prior.pi.low.rct = NULL,*

*prior.pi.high.nrs = NULL,*

*prior.pi.low.nrs = NULL,*

*## ---------- when method.bias = "prior" -------*

*run.nrs.var.infl = 1,*

*run.nrs.mean.shift = 0,*

*run.nrs.trt.effect = "common",*

*run.nrs.n.adapt = 1000,*

*run.nrs.n.iter = 10000,*

*run.nrs.n.burnin = 4000,*

*run.nrs.thin = 1,*

*run.nrs.n.chains = 2,*

*##*

*backtransf = gs("backtransf"),*

*##*

*run.nrs.n.thin = NULL)*

To begin, we should provide the following variables: treatment arm *trt*, *study* id, observed *outcome*, sample size *n* (only for AD studies), study *design and* standard error *se* (needed only for AD studies with continuous outcome). These variables are the names of the columns in participant data (*prt.data*) and aggregate data (*std.data*). It is important that the corresponding columns in *prt.data* and *std.data* should have the same name. The summary measure should be indicated by *sm,* which can be *"OR"* (Odds Ratio), *"RR"* (Risk Ratio), *"MD"* (Mean Difference) or *"SMD"* (Standardised Mean Difference). Network reference treatment can be assigned to *reference*, otherwise the first alphabetic treatment will be used as a reference in the analysis.

To perform an NMR, we should add the covariates (up to 3) to (*cov1*, *cov2* and *cov3*). To enhance model run efficiency, we centre each continuous covariate around a value set as *cov1.ref, cov2.ref* and *cov3.ref*. The default for these values is the overall minimum of covariate entries from all studies. The *split.regcoef* argument determines whether split the within- and between-covariate coefficients $\beta_{1,jbk}^{W}$ and $\beta_{1,jbk}^{B}$ are split. We recommend estimating these two parameters separately, so we make it the default (*split.regcoef=TRUE*).

We can also compute the values of Surface Under the Cumulative Ranking (SUCRA) (by enabling the sucra=TRUE option), but it's essential to specify a negative preferred direction for the outcome (using small.values="desirable"). This setting indicates that lower values of the relative treatment effect signify the treatment's effectiveness. Conversely, if positive values are preferred, you can set small.values="undesirable". Then the arguments *cov1.value, cov2.value and cov1.value* are the participant covariate values for which to report SUCRA results for *cov1*, *cov2* and *cov3*, respectively. These arguments should be set for NMR, sucra=TRUE and when individual participant dataset is used in the analysis. For dichotomous covariates, a character of the level (used in the data) should be indicated.

Set *method.bias="naive"*, *"prior"*, *"adjust1"* or *"adjust2"* to specify one of the four cross NMA/NMR models. When *method.bias="adjust1"* or *"adjust2"*, the following columns should be present in both datasets: *bias*, *unfav* and *bias.group*. Only if *bias.group* is missing, default values are assigned to it. The bias effect can be added to the relative treatment effect (*bias.type="add"*, default), multiplied (*bias.type="mult"*) or both (*bias.type="both"*).

The bias probabilities $\pi_{j}$ are either assigned beta distributions with the following default values: RCT with low RoB (prior.*pi.low.rct="dbeta(1,20)"*) and high RoB (prior.*pi.high.rct="dbeta(20,1)"*), NRS with low RoB (prior.*pi.low.nrs="dbeta(1,30)"*) and high RoB (prior.*pi.high.nrs="dbeta(30,1)"*). Users are encouraged to provide their own beta distributions (through *prior* argument) in order to assess the results’ robustness to the assigned distribution. Alternatively, $\pi_{j}$ can be predicted using a specific study characteristic which should be supplied to *bias.covariate*.

We can assume independent, random-effects, or common-effects to synthesize the effects across studies (correspond to arguments ends with *.effect*). The options for each parameter are summarized in Table 1.

When NRS data is used as a prior information (*method.bias="prior"*), several arguments can control the process, all of which begin with *run.nrs*. These are the arguments: *run.nrs.var.infl* controls the inflation of the variance of NRS estimates, and its values range from 0 (NRS contribute nothing and the prior is vague) to 1 (the NRS evidence is used at face value, default approach). The parameter *run.nrs.mean.shift* represents the bias shift to be added/subtracted from the estimated NRS mean treatment effects (0 is the default). Users can also specify the arguments to control the MCMC chains (used to run NMA/NMR for NRSs) with the default value in the parentheses: the number of adaptation *run.nrs.n.adapt* (1000), the number of iterations *run.nrs.n.iter* (10000), number of burn in *run.nrs.n.burnin* (4000), number of thinning *run.nrs.thin* (1) and number of chains *run.nrs.n.chains* (2).

The arguments start with *.prior* lets users specify their own prior distributions for several parameters. When effects are set to *"random"*, the user can provide the between-study heterogeneity parameters for as follows: prior.*tau.trt* for the treatment effects, prior.*tau.reg0* for prognostic covariate effect, prior.*tau.regb* and prior.*tau.regw* for within- and between-study covariate effect, respectively, and prior.*tau.bias* for bias effect.

Finally, the argument *backtransf* indicates whether results should be back transformed in printouts. If backtransf = TRUE, results for sm = "OR"} are presented as odds ratios rather than log odds ratios, for example.

## Run cross NMA/NMR model

To run the model generated from *crossnma.model(),* call the *crossnma()* function in R as follows:

*crossnma(x, n.adapt = 1000, n.burnin = floor(n.iter/2),n.iter = 2000, n.thin = max(1, floor((n.iter - n.burnin) / 1000)), n.chains = 2, monitor = NULL, level.ma=x$level.ma, backtransf=x$ backtransf, quiet=TRUE)*

*crossnma.model()* returns x, which is used to run the MCMC methods and estimate the parameters. The MCMC chains run under the following settings, with the default value in the parentheses: the number of adaptation *n.adapt* (1000), number of iterations *n.iter* (2000), number of burn in *n.burnin* (floor(n.iter / 2)), number of thinning *thin* (max(1, floor((n.iter - n.burnin)/1000))) and number of chains *n.chains* (2). However, it is recommended that these values be set based on the Monte Carlo (MC) error, which take into account the number of iterations and the level of autocorrelation, so that MC error does not exceed 5% of the posterior standard deviation.

The argument *monitor* takes a vector containing the names of the parameters to be monitored. To find the spelling of these names, the user can check the JAGS model (*jagsmodel*) produced as an output from *crossnma.model()*. In general, users should inspect the generated *jagsmodel* before running the analysis to ensure that the specified arguments in *crossnma.model()* have created the desired model.

The argument *level.ma* sets the level used to calculate credible intervals for

network estimates and *backtransf* indicates whether results should be back transformed in printouts. The default values for these arguments are inherited from the the *crossnma.model()* function*.*

## Display the findings of the analysis

**Net plot**

The *netgraph()* function can visualize the network of evidence by calling the *netgraph()* function from *netmeta* package internally. This function takes the output of *crossnma.model()* as input and generates a graph with nodes representing available treatments and edges connecting any two treatments if at least one study compares them directly. This graph is useful for visually checking network connectivity. This is especially important in Bayesian settings, because when data for disconnected networks is missing, priors are not updated, and the analysis will only provide estimates as prior information.

The *crossnma()* function will create the posterior distribution for each parameter, and the functions that follow will display the results in tabular or graphical form.

**Summary table**

The generic function *summary()* summarizes the mean, standard deviation and the 2.5%, 50%, 97.5% percentiles of the posterior distribution. It also provides two statistics to assess convergence $\hat{R}$ (*Rhat*) and the number of effective sample size (*n.eff*). The Gelman-Rubin statistic $\hat{R}$ quantifies variation within and between chains. When chains converge properly, $\hat{R}$ should be close enough to one. The number of effective sample size indicates the number of iterations used to construct the posterior distribution. This value should be sufficiently close to the number of iterations (after discarding the burn-in).

**Trace plot**

This function plots the simulations from each iteration and connects the points with lines. The generated plot is used to visually check the convergence of each parameter, allowing the user to inspect if the different chains mix well and approach to a specific value.

**League table and heat plot**

The *league()* function creates a table containing the median (or mean) as well as the lower and upper bounds of the 95% credible interval of the relative effects for each possible pair of treatments. The *heatplot()* function displays the same information but in graphical formats. It creates a matrix where the entries in each cell show the median and the 95% credible interval of the relative effects of the treatment at the top versus the treatment on the left.

In both functions, the number of digits used to display the results can be specified to *digits.* The user can control also the order of the treatments by providing a vector of the treatment names to *order* and this will be the order in which the treatments are displayed. If the user wants to show mean estimates, set the argument *median* to *FALSE;* otherwise, the median will be produced by default. The argument *exp* enables exponentiate the values presented, which is useful when analyzing binary data because the analysis is done on the log-scale but the summary measure is better expressed in terms of odds ratio or relative risk*.*

When the NMR model is fit, the relative treatment effects for the covariate values *cov1.value*, *cov2.value* and *cov3.value* are shown. If users want to change those variables, they have to do so from the start when they build the model using *crossnma.model()*.

The league table can be created in *“wide”* or “*long*” formats, which is to be specified to *direction.* The user can modify the settings of the heat plot by customizing the text size using *size*, *size.trt* and *size.axis,* and the colour of the cells through *low*.*colour*, *mid.colour* and *high.colour.*
