# Supplementary material for: crossnma: An R package to synthesize cross-design evidence and cross-format data using network meta-analysis and network meta-regression
Source: BMC Med Res Methodol. 2024 Aug 5;24:169. doi: 10.1186/s12874-023-02130-0 (PMC11299362; doi:10.1186/s12874-023-02130-0)
Supplement: Supplementary file 11 — Additional file 11. Reviewer reports submitted for version 1. [file 12874_2023_2130_MOESM11_ESM.docx]

**Reviewer reports submitted for version 1**

**Reviewer 1**

Submitted: **18 Apr 2023**

The paper focuses on introducing the R package 'crossnma' for conducting Bayesian Network Meta-Analysis (NMA) and Network Meta-Regression (NMR) using the JAGS software. The package is designed to handle complex data structures, including different study designs such as randomized controlled trials (RCTs) and non-randomized studies (NRS), as well as individual participant data (IPD) and aggregate data (AD). The paper is well-structured, with an introduction that explains the need for such an R package and highlights the current state of NMA and NMR. It provides a brief background on NMA and NMR and their potential applications, followed by a description of the crossnma package's features and functionality. The authors then compare the output of crossnma with BUGSnet using a dataset and discuss the differences in the results obtained. The working example section demonstrates the use of crossnma for various analyses, including unadjusted NMA, unadjusted NMR, using non-randomized studies as a prior in NMR, and bias-adjusted models. The authors provide an R code for each analysis, illustrating the package's usability and flexibility. The discussion section acknowledges some of the package's limitations, such as the lack of support for count and time-to-event outcomes, and the need for additional result presentation methods. The authors plan to address these issues in future versions of the package.

The paper has several strengths:

1. The use of Bayesian methods for network meta-analysis and network meta-regression provides a flexible and coherent statistical framework for synthesizing data from different study designs and formats (RCTs, NRS, IPD, AD). This allows the researchers to incorporate various sources of information and uncertainty in the analysis.

2. The crossnma package uses the JAGS software for MCMC sampling, a well-established tool for Bayesian analysis, which adds credibility to the underlying methodology.

3. The paper provides a comprehensive working example, demonstrating how to use the package for various types of analyses, including unadjusted NMA, unadjusted NMR, using non-randomized studies as a prior in NMR, and bias-adjusted models. This is useful for researchers intending to use the package in their work.

4. The authors compare the results of the crossnma package with another established package, BUGSnet, using a dataset. This comparison helps establish the reliability of crossnma in terms of generating accurate and meaningful results.

Although the authors express a few limitations in their paper, but there are a few more weaknesses and limitations worth noting:

1. The package currently supports only binary and continuous outcomes, limiting its applicability to certain types of research questions that involve count or time-to-event outcomes. (though the authors do express their intension to expand the package’s ability to analyze count and time-o-event data, which is quite exciting).

2. The data input format is restricted to arm-level data, and the package does not allow for direct input of contrast-level data. This could require additional data manipulation efforts for some users.

3. Inconsistency assessment methods, such as node splitting and unrelated mean effect, seem to not yet be implemented in crossnma. Inconsistency is an important aspect of NMA and NMR, and its evaluation should be incorporated into the package.

4. The paper does not provide a comprehensive evaluation of the package's performance in terms of computational efficiency and scalability, especially for large or complex datasets. It would be beneficial to assess the package's performance under various scenarios to ensure its suitability for different research applications.

5. The paper could benefit from a more detailed discussion of the underlying statistical assumptions and potential biases that may arise when using crossnma, especially when combining data from different study designs and formats.

6. It would help to include a more comprehensive literature review to contextualize the crossnma package within the broader landscape of NMA and NMR tools and methodologies.

A few other points of note:

• From a linguistic perspective, the following is my assessment of the assessment of writing quality, understanding easiness, clarity, and organization.

• The paper could benefit from a more detailed discussion of the limitations of the crossnma package, including any potential pitfalls or biases that users should be aware of when using the package.

• The authors could provide additional examples or case studies to demonstrate the package's applicability and performance in a variety of research contexts.

Overall, the paper presents a valuable contribution to the field of NMA and NMR, as it introduces a flexible and user-friendly R package capable of handling complex data structures. The paper is well-written, with a clear focus on the package's functionality and potential applications. The working examples and R code provided throughout the paper make it easy for readers to understand how to use the package effectively in their research.

**Reviewer 2**

Submitted: **25 Apr 2023**

REVIEW: Hamza et al: crossnma: An R package to synthesize cross-design evidence and cross-format data using network meta-analysis and network meta-regression

Summary

This paper presents an R package that can carry out 4 kinds of network meta-analysis (NMA) analyses, described as

(a) “unadjusted synthesis of RCTs and non-randomised studies (NRS)”

(b) an NMA of RCT data with informative priors on relative treatment effects based on the NRS

(c) an analysis with relative treatment effects “adjusted” using information from Risk of Bias (RoB) assessment

(d) an analysis like (c.) but with a different method of bias adjustment. An important feature is that all four analyses are intended to combine aggregated data (AD) and individual patient data (IPD), while taking advantage of the IPD to ‘adjust’ for effect-modifying covariates. Of particular relevance: the authors intend for the work to support health technology assessment and decision making.

The full details of the models whose implementation in R is described here appear in reference (15) by the same authors, a document in the arXiv repository and therefore not peer-reviewed. I have had to consult their earlier paper to better understand their proposals and their rationale. The arXiv paper has now been published in Research Synthesis Methods, but I have not checked to see if this differs.

Major comments

Rationale for inclusion of NRS

The authors mention “growing concerns” about RCTs. I have not encountered this myself, but I have heard growing concerns about the increasing use of NRS in HTA. The authors cite the fact that most trials use placebo comparators. It’s not clear how this justifies the use of NRS data, which are inherently prone to multiple biases of unknown magnitude. Choice of comparators is not random, of course, but choice of comparator cannot in itself result in biased estimates.

In reference (15) the authors also cite Anglemyer (2014) to the effect that, on average, RCT and NRS studies produce very similar results. This is irrelevant: it overlooks the fact, reported in the same paper, that the heterogeneity in the RCT: NRS odds ratio was “substantial”. This means that, on the contrary, in any given case, namely in the situation faced by re-imbursement or clinical guideline committees, one would expect substantial differences between treatment effects estimated in NRSs and RCTs.

The authors also claim that RCTs are vulnerable to sponsor bias. My understanding is that sponsors may game the system through their choice of comparators, but (see above) this does not introduce bias is their estimates. Sponsor bias is not included in the approved Risk of Bias tool. The fact that industry trials tend to show that active treatments are better than placebo is not evidence for biased estimates. The few studies that are properly designed to reveal bias in estimates in favour of the sponsor’s product have failed to find it.

Method for combining AD and IPD

1. The first equation in section 1.2.1 (page 5) set out the model for the probability of response to treatment k in trial j, for an individual i in IPD. The second equation gives the probability of response in AD. The same relative treatment effect delta(j,b,k) relative to baseline treatment b appears in both equations. These equations cannot both be correct. The relative treatment effect in a standard AD meta-analysis cannot be the same as the relative treatment effect estimated in an IPD analysis, unless linear predictors are on collapsible scales. A logistic link function is used throughout, so the equations would be incorrect even if there were no effect modifiers.
   The authors cite papers by Scaramago and Donegan, which make the same incorrect assumption, and also by Jensen, which does not. Instead, the response at the aggregate level can be found by integrating over the distribution of covariates (Jackson 2006, 2008; Jensen 2012; Phillippo 2020,2022). As the logistic transformation is non-linear it is not enough to rely on the mean covariate value in the model for AD.
   Possibly, although I would not advise this, the authors could describe their model as an approximation to what would be obtained by proper integration. If so there would need to be some commentary on how accurate this approximation would be under realistic assumptions about the variance of covariate distributions.
2. The authors do not address how the output from the combined AD and IPD synthesis will be used for decision making. Now that the model incorporates treatment-by-covariate interaction terms, it follows that the differences between alternative treatments will depend on the precise covariate mix in the population targeted for decision. There is no mention of ‘target’ or ‘population’. It is evident from the examples (page 17) that the authors intend to plug in target values of covariates, again ignoring the variance. The final relative treatment effects are therefore relevant only to an individual with specific covariate values, not to any actual population. In addition, there is no estimation of (absolute) population average responses under each treatment, making it hard to see how the proposed software could be used for HTA.
3. The models allow effect modifying covariates to work in a range of ways, including having a different regression coefficient for each treatment. In typical datasets used for HTA, which can be quite sparse, it is unlikely that such models would be estimable unless there was IPD on each treatment. A more serious problem, however, is that in an HTA context, stakeholders would be unlikely to accept that each treatment was associated with a different (effect modifier) regression coefficient. Decision makers are reluctant to contemplate effect modifiers without strong evidence, and different effect modifiers for each treatment seems highly implausible. It would be more plausible if all treatments, or at least all treatments within the same class, were considered to have the same regression coefficients. Authors should provide, or refer to, guidance on how users of their software should proceed.

Bias-adjusted models

1. NRS priors: This method incorporates relative treatment effect information from NRS as priors in a subsequent standard analysis of RCT data. (This is in fact approximately equivalent to incorporating both RCT and NRS in the same analysis). Obviously, all this achieves is to introduce an unknown level of bias into the final estimate. No Bayesian trialist would accept the use of priors derived so crudely from NRS. The authors suggest that “to limit the impact of NRS on RCT estimates” the prior variance can be inflated, or expert opinion could be consulted. Arbitrary and/or post hoc manipulation of the weight given to NRS does not meet the level of methodological transparency and consistency required for HTA methods, and manufacturers are unlikely to accept them. The other alternative is expert opinion. The authors cite Turner (2009),which is is an extremely detailed and convincing example of expert analysis of bias, but one that required a panel of outstandingly knowledgeable experts and 5 hours per expert per study. As far as I know this method has hardly ever been used in practice.

2. Bias-adjustment model 1. Here they employ Verde’s (2021) extension of bias-adjustment models of Dias (2010). Verde's method includes both a term for bias and a term for the probability of bias. The authors need to clarify under what circumstances it is possible to identify both these parameters as well as other parameters control variances, from the data alone. At first sight it seems that, unlike the Dias model, which leverages the NMA structure to estimate the bias parameter only from the data at hand and without strongly informative priors, the Verde extension requires informative priors to produce results. If so, it will be criticized on the same grounds at the NRS priors approach – that it lacks transparency and is not suitable for decision making. (See comments on Results). The default priors for the probability of bias parameter (page 6), beta(10,1) for “high risk” RCT, beta(30,1) for high risk NRS), for example, suggest that the method relies on highly informative, yet arbitrary and non-evidence-based, priors.

3. Bias-adjusted model 2. Once again, authors should tell investigators who are considering using this model under what circumstances the parameters can be identified by the data alone. That is, without arbitrary, investigator-originated priors. Otherwise, in a re-imbursement or clinical guideline context, treatment recommendations will depend on arbitrary guesses.

4. There are methods for correcting the bias in NRS, that are entirely data-driven and do not rely on strong informative priors - or guesses - regarding the extent or direction of bias. These methods are not among those recommended in this paper, which may severely limit the applicability of this software in practice.

Results of applying the models

Results of applying the models to an illustrative data ensemble are shown in Table 3. It would be helpful if, in section (c) of the table, the relative treatment effect estimates could be made relative to treatment A, to aid comparison to the other sections.

In section (d) the high level of posterior uncertainty in the mean bias parameter, “g”, suggests that it was very poorly estimated; or even that there was no contribution at all from the likelihood. Authors should check this, or at least comment. Could the poor estimation of this parameter be due to the configuration of the bias model (see above) in which there are parameters for both the probability of bias and the extent of bias, leading to an identifiability problem?

Perhaps more important is the rationale for including this parameter in the model. In this case the probability of bias is linked to the year when the study was carried out. Readers familiar with decision making in HTA might wonder how such a model can be justified. It is hard to imagine that manufacturers whose products were trialed earliest would accept that their studies were more biased, and that estimates of their product’s efficacy should be more down-rated. Apparently the worked examples are based on fictitious data. This is acceptable in itself, but the examples chosen create a poor impression.

Assumptions

It would be useful to clarify the assumptions being made, particularly as these are not fully described in the methodological paper (15). For example, the method for combining IPD and AD (like all other methods) it assumes that there are no unidentified effect modifiers.

A full list of assumptions would be useful, even if in an appendix.

Minor comments

1. Last line of abstract: “… facilitates inclusion of all types of evidence accounting for their differences in risk of bias”. I don’t think it is correct to say that such differences are “accounted for”? Better to use a word like “recognising differences ….” ?

2. “growing concerns” about RCTs (page 2). This is a somewhat journalistic way of expressing what is no more than a personal view. Authors should confine themselves to citing and commenting on literature. (Although see above for comments on their interpretation of the literature)

3. The equations (page 5) seem to allow for only a single covariate, but the software seems to be capable of handling more than one covariate. Can the authors clarify whether or not the model and software are restricted in this way, and adjust the equations accordingly

4 The second equation on page 5 has “+ +” in it.

5. There are a couple of error messages relating to the references

6. Page 18: “number of effective samples sizes” …meaning “effective sample size”?

7. The trace plots (Fig 4) don’t seem very helpful. Density plots would be a better way of showing that stable posteriors had been achieved.

8. Page 20: “there is a grow…”

9. Page 20: “However, data is increasingly becoming available in a variety of formats … and only user-written code can be used …”. Contrary to what is implied in this section, crossnma is not the only package capable of combining IPD and AD: multinma is another, cited in Phillippo (2022). Multinma relates treatment effects at IPD and AD levels via integration over the covariate distribution.

**Reviewer 3**

Submitted: **26 Apr 2023**

This paper presents an R package crossmeta to synthesize cross-design evidence and cross-format data using network meta-analysis and network meta-regression. The methods and results are relatively well presented, and the relevance is clear.

1. Graphical tools: The network plots are not sophisticated. Can you make more informative graphs that reflect the sizes of statistical information in nodes and edges (like the network package of Stata).

2. The authors noted “The individual participant data (IPD) of the study, when available, are preferred to AD for adjusting for important participant characteristics and to better handle heterogeneity and inconsistency in the network.” in abstract. However, there are no tools for evaluating inconsistency in the crossmeta package. Please add the standard tools to evaluate the inconsistency.

3. Please add the ranking evaluation tools such as SUCRA.

4. In the Applications section, the authors adopted odds-ratio as the effect measure. However, it is inadequate for evaluating treatment effect (please see Cochrane Handbook). Risk-ratio or risk-difference is preferable.

5. Please explain clearly what are the advantages of the crossmeta methods comparing the ordinary two-stage synthesis method. Also, please compare the two approaches in the Application section to show the advantages (if possible).

**Reviewer 4**

Submitted: **28 Apr 2023**

This manuscript introduces the R package “crossnma” developed by the authors for combining evidence across different designs and formats. In general, it is written well from my perspective. The manuscript provides a good resource for researchers to synthesize IPD and AD from RCTs and NRS using a working example with sample codes. I only have some minor comments, as follows.

On page 5, the model for level 2 contains two plus signs.

Page 6, section 1.2.3 mentions “model 1.1”, though the models are not numbered in this manuscript.

Section 2.2 compares crossnma with BUGSnet based on the working example. Section 4 mentions two more R packages gemtc and bnma, which also use JAGS for the Bayesian framework. It might be helpful if the authors could discuss more on the comparisons among these packages, such as the reason why comparing crossnma with BUGSnet instead of getmc. To me, the package's most attractive part is enabling evidence synthesis across sources and formats, not for advantages of the Bayesian framework. I would like to learn about the benefits of conducting cross-format and/or cross-design NMA and NMR via crossnma compared to other packages. Authors might want to explore those packages/tools that combine evidence of IPD and AD levels, or evidence from RCTs and NRS, e.g., multinma for ML-NMR by Phillippo et al. 2020 (<https://www.ncbi.nlm.nih.gov/pmc/articles/PMC7362893/>).

Page 11, although section 3.1 states that the IPD dataset contains “2950 rows”, dataset “ipddata” used in the supplementary information 6 only has 1944 rows in total.

Page 11, the MCMC setting with 5000 iterations, 2000 burn-in, and one thinning in section 3.2 is very confusing, as it differs far from the default MCMC settings (say, 100000 iterations, 40000 burn-in and thinning rate of 5) described in other sections.

**Reviewer 5**

Submitted: **29 Apr 2023**

This paper introduced an excellent R package facilitating the combination of aggregate data (AgD) and individual data (IPD), as well as randomised trials and non-randomised studies of intervention. This package will be very helpful and fit many studies meeting the issue. I have only some minor issues for discussion.

1. The package adjusted the ecologic fallancy in combining AgD and IPD by splitting the interaction into between-study and within-study and linking the study-level mean and individual-level effects. An alternative approach involves integrating the individual level model over the covariate distribution to create an aggregate level model. In section 2.2, super if the manuscript compares the two approaches (e.g., using multinma package) instead of a traditional Bayesian approach by BUGSnet.

2. For readers with non-statistical background, it could be helpful to clarify the prior and model structure assumptions in Table 2.

3. In Section 2.1, starting with a description of the input data format may be helpful.

4. In section 3.1, a figure illustrating what the data request could be helpful.

**Recommendation for version 1**

**Editor: Fares Alahdab**

Submitted: **02 May 2023**

Recommendation: *Request Revision*

Thank you for submitting your manuscript to our journal for consideration. We appreciate the time and effort you have put into this research. We have completed the initial review of your manuscript. The topic is relevant and has the potential to contribute valuable insights to the field. However, based on the critiques received from our reviewers, we kindly request that you revise your manuscript to address them. Once these revisions have been made, please resubmit your manuscript for further consideration. We believe that addressing these concerns will help to strengthen your paper and its potential impact on the field. We look forward to receiving your revised manuscript. If you have any questions or concerns, please do not hesitate to contact us.
